# Self-Powered Active Sensor with Concentric Topography of Piezoelectric Fibers

**DOI:** 10.1186/s11671-016-1786-x

**Published:** 2017-01-17

**Authors:** Yiin Kuen Fuh, Zih Ming Huang, Bo Sheng Wang, Shan Chien Li

**Affiliations:** 1 Department of Mechanical Engineering, National Central University, No.300, Jhongda Rd., Jhongli District, Taoyuan, 32001 Taiwan (R.O.C.); 2 Institute of Materials Science and Engineering, National Central University, Taoyuan, Taiwan

**Keywords:** Piezoelectric generator with concentrically circular topography (PGCT), Direct-write, Near-field electrospinning (NFES), Polyvinylidene fluoride (PVDF), Deformation sensors

## Abstract

In this study, we demonstrated a flexible and self-powered sensor based on piezoelectric fibers in the diameter range of nano- and micro-scales. Our work is distinctively different from previous electrospinning research; we fabricated this apparatus precisely via near-field electrospinning which has a spectacular performance to harvest mechanical deformation in arbitrary direction and a novel concentrically circular topography. There are many piezoelectric devices based on electrospinning polymeric fibers. However, the fibers were mostly patterned in parallel lines and they could be actuated in limited direction only. To overcome this predicament, we re-arranged the parallel alignment into concentric circle pattern which made it possible to collect the mechanical energy whenever the deformation is along same axis or not. Despite the change of topography, the output voltage and current could still reach to 5 V and 400 nA, respectively, despite the mechanical deformation was from different direction. This new arbitrarily directional piezoelectric generator with concentrically circular topography (PGCT) allowed the piezoelectric device to harvest more mechanical energy than the one-directional alignment fiber-based devices, and this PGCT could perform even better output which promised more versatile and efficient using as a wearable electronics or sensor.

**Electronic supplementary material:**

The online version of this article (doi:10.1186/s11671-016-1786-x) contains supplementary material, which is available to authorized users.

## Background

Due to the rise of huge demand for portable or wearable electronics, the self-power system is deemed to be indispensable for the ubiquitous computing systems. Piezoelectric materials provide a feasible way to effectively harvest energy from ambient sources or human actions [1–7] rather than depending on cell batteries. The piezoelectric properties have been studied broadly since 2006, the first piezoelectric energy harvester constructed by zinc oxide (ZnO) nanowires (NWs) arrays [8] have been developed as a promising and new power sources which could convert mechanical energy to electric energy [9–13]. The amazing debut inspired great interests for developing further applications based on piezoelectric materials. In order to catch up with the booming market of portable smart electronics, the batteries should be ultra-light, small, eco-friendly and sustainable. However, batteries research still struggled with many confinements and the piezoelectric nanogenerator (NG) [14–19] could be an alternative way to meet the ever-increasing need of energy. Besides ZnO NWs, a lead zirconate titanate (PZT) NWs NG [20–22] was presented to scavenge the mechanical energy too and the output voltage and power could reach to 1.63 V and 0.03 μW [23]. Another research on piezoelectric materials is utilizing hydrothermal method to synthesize BaTiO_3_ nanotubes and the measured output could be even higher which reached to 5.5 V and 350 nA [24]. Yet, theses mentioned piezoelectric systems needed exacting and fussy processes to fabricate, such as bottom-up assembly or high temperature sintering and post-poling. Consequently, electrospinning technique is comparatively simple, economical and versatile process to fabricate nano/micro fibers (NMF)-based piezoelectric NG [25–28] from polymers or composites materials. A popular piezoelectric polymer, polyvinylidene fluoride (PVDF), has been studied widely due to its highly stretchable flexibility, biocompatibility, and cheap expense [29–31]. The recent study presented controllable, direct-write and patterning manners via near-field electrospinning (NFES) which used PVDF as main piezoelectric material [32, 33]. The major virtue of NFES PVDF fibers is the larger piezoelectric strain constant (d_33_ ∼−57.6 pm/V) and energy conversion efficiency (eta ~20%) [34] compared with traditional PVDF thin films (d_33_ ∼−15 pm/V, eta is less than ~5%) [35]. This significant result was primarily contributed due to PVDF’s semi-crystalline structure, the β crystalline phases which is responsible for the enhancement of piezoelectric property [36, 37]. The dipole moments of β phased are pointing in the same direction which could be obtained from electrospinning. In order to massively deposit highly aligned and polarized fibers, NFES would be a good candidate due to the inherent nature of simultaneous induction of mechanically stretching and electrically poling process. In addition, a massively parallel aligned 500 micro-fibers based PGCT deposited via oriented poled and in situ NFES has successfully produced a peak output voltage of 1.7 V and current of 300 nA in the recent study [38]. To summarize these aforementioned features of NFES PVDF, the applications for energy conversion have been demonstrated in a diverse variety of areas, such as electromechanical actuators, self-power systems, and active sensors for rehabilitation application [39–43]. Here, we demonstrated spider web inspired PGCT based on NFES PVDF fibers with the concentric circle pattern. The distinctively unique topography makes it more feasible to harvest mechanical energy from different bending direction. In comparison, this versatile functionality is not attainable for parallel aligned piezoelectric fibers such that the power can only be scavenged by bending direction which is closed or parallel to aligned direction of piezoelectric dipoles. This modification not only made the PGCT workable under differentially deformed direction but also had a fine output voltage (~2.5 V) and current (150 nA) with a rotating cantilever flapping test and furthermore, human motion detection of palm, wrist, and elbow motions.

## Methods

**Fig. 1:**
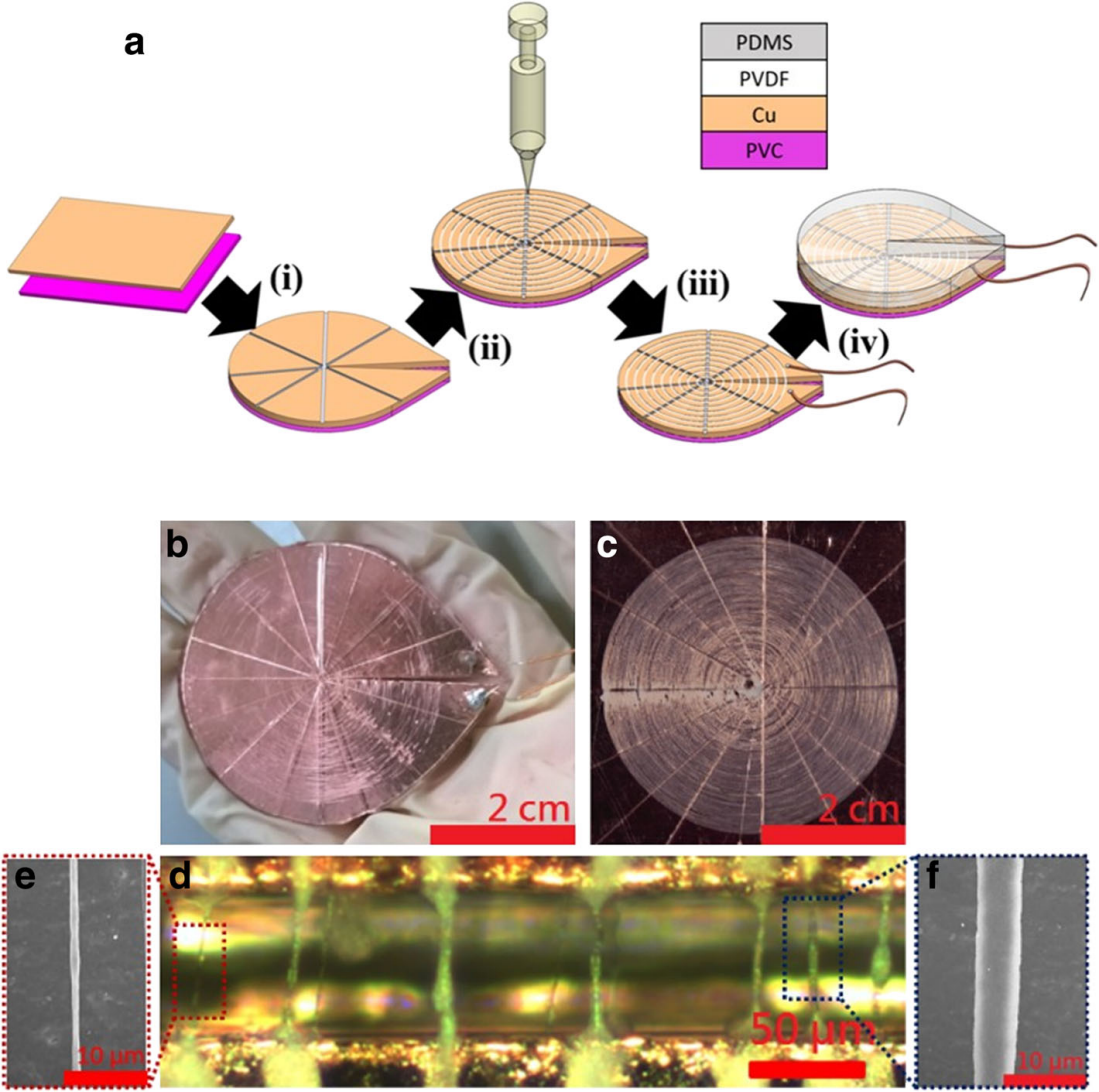
**a** Schematic of the near-field electrospinning process to fabricate direct-write PVDF fibers with concentric circle topography. (*i*) Added gaps in Cu foil and cut with Polyvinylchloride (PVC) substrate into proper shape. (*ii*) Fabricated PVDF fibers via NFES. (*iii*) Welded wires on Cu foil. (*iv*) Fully encapsulated with Polydimethylsiloxane (PDMS). **b** Photographic image of the finished device. **c** PVDF fibers deposited on the copper foil before encapsulated. **d** Optical microscope image of the fabricated fibers. **e**, **f** Enlarged SEM photomicrographs showed a single PVDF fiber

The piezoelectric generator with concentric circle fibers have been demonstrated in this article and the fabrication process consists of four steps as shown in Fig. 1a.

The schematic diagram illustrates the pivotal processes in fabricating the concentric circle fiber-based PGCT. Initially (i) the Cu foil was glued on the PVC substrate and added gaps in the Cu foil with the razor blade then cut into desired shape (diameter about 4 cm). After that, (ii) the PVDF piezoelectric fibers were continuously deposited on the Cu foil electrode via in situ poled NFES technique (needle top to Cu collector distance ~1 mm) which has the great controllability to pattern the fibers into concentric circle. In process (iii), the Cu wires were soldered on two end sides separately. In order to protect the piezoelectric fibers and make the structure more robust, (iv) the final packaging step is utilized PDMS to fully encapsulate. The photograph in Fig. 1b shows the piezoelectric generator with concentric circle fibers was fabricated by a simple and cost-efficient process. The four layers (PDMS, PVDF fibers, Cu foil, and PVD substrate) pliable structure also enabled the PGCT to demonstrate great flexibility. And, the PGCT consists of ~100 NMFs which were precisely deposited into concentric circles on the Cu electrode as shown in Fig. 1c. In addition, the gaps in the Cu foil which could separate more numbers of electrodes and the fibers were totally suspended when crossed the gap. This phenomenon would make the piezoelectric fibers to scavenge mechanical energy more efficiently and the electrodes placed between fibers could simultaneously enhance the output performances due to the electrical superposition effect of in serial/parallel connection was obtained. In Fig. 1d, the optical image of the fabricated fibers was electrospun on the Cu electrode with the working gap between two electrodes being ~50 μm. The diameters of as-spun PVDF fibers might range from hundreds nm to several μm due to the spinnability of PVDF solution. The continuous deposition of PVDF NMFs was fabricated under restricted operating region at the sacrifice of diameter variation of NMF which was identified in previous research [38]. Figure 1e, f shows the scanning electron microscopy (SEM) images of two intentionally chosen PVDF NMFs with notably different diameters which were both fabricated via direct-write NFES. The characterization result of the non-uniform fiber diameter (in the range of nano-to-micro scale) as fabricated via NFES technique indicates the tradeoff between the continuous spinnability and uniformity of electrospun fibers.

**Fig. 2:**
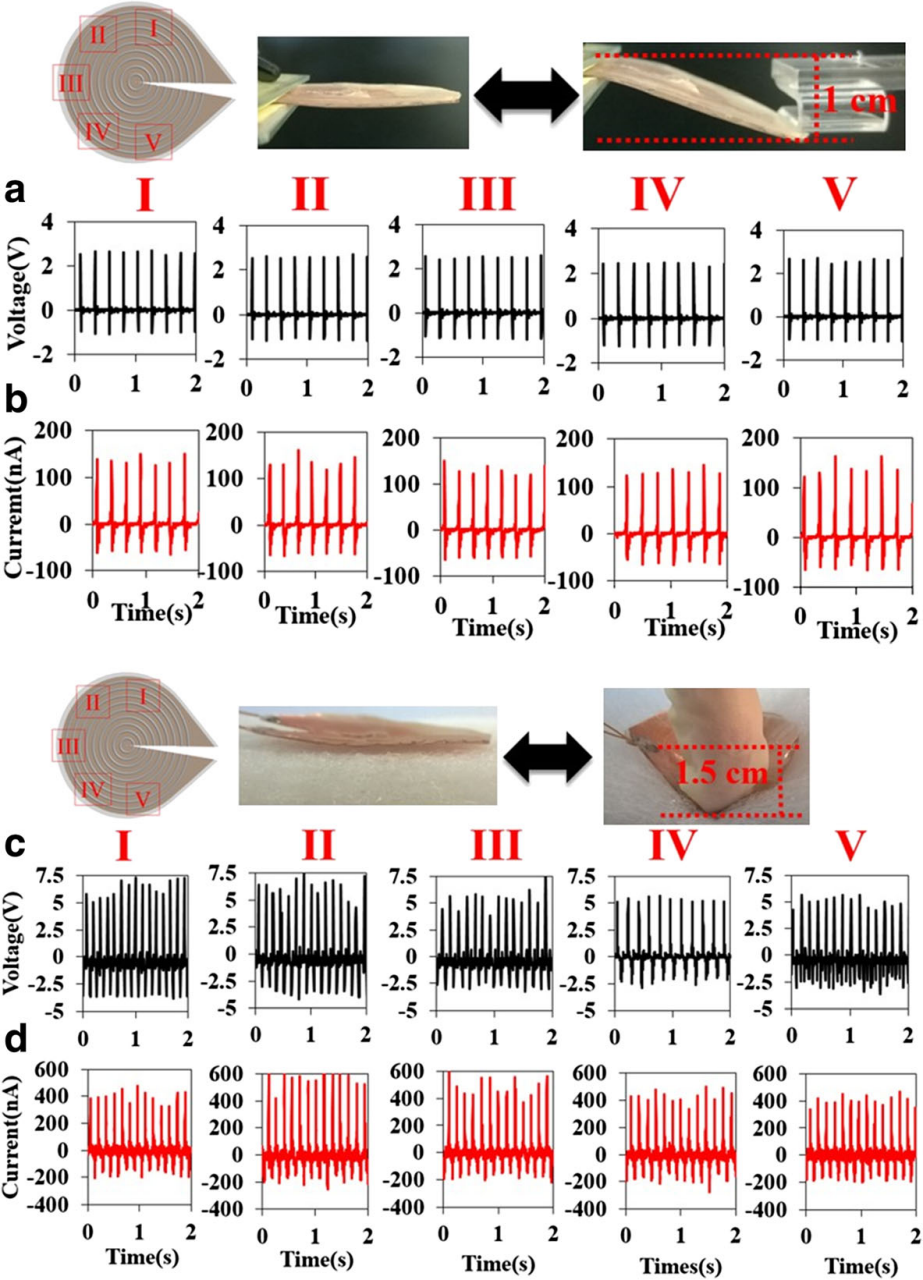
Measured output voltage and current. Voltage (**a**) and current generated by flapping the corresponding position at constant frequency of approximately 4.5 Hz (**b**). Placed the PGCT on the cotton fabric and pressed the corresponding position to obtain open-circuit (**c**) voltage and (**d**) short-circuit current

The PGCT with concentric fibers was carried out to improve the ability of collecting mechanical energy from different directions. Compared to the traditional NG with parallel aligned fibers which cannot harvest energy in specific movements, such as bending along or closed to the poling direction. This distinctive characteristic of the concentric fibers based PGCT demonstrated a promising future in sustainably harvesting minute motions into valuable energy without any restriction. In Fig. 2a, we investigated the performance of the PGCT by flapping on the different positions at constant frequency of approximately 4.5 Hz. The details of flapping experiment layout is shown in Additional file 1: Figure S1. Here, we randomly chose five positions (I, II, III, IV, and V) of the PGCT to test the output voltages and currents. The results showed that the average output voltages/currents were 2.5 V and 150 nA, respectively. The major purpose was also achieved, which the output magnitude of both voltage and current were similar in different operating positions, showing the capability of harvesting mechanical deformation in arbitrary direction. Furthermore, in Fig. 2c, d, the finger induced deformation based on five positions (I, II, III, IV, and V) of the PGCT and the related output performance is presented as a comparison with Fig. 2a, b. The PGCT was settled on the cotton fabric and pressed by a finger which had the average output voltage/current of about 5 V and 400 nA. The output magnitude between each position was approximately same which again exhibited the great accommodation to scavenge mechanical energy from different actuated direction. The electrical signals were monitored from an oscilloscope and the output signals in Fig. 2b are obviously larger than Fig. 2a. This observed result was primarily attributed to an the larger displacement (~1.5 cm) than the flapping test (~1 cm) was created on the PGCT in the finger pressing test which resulted in the higher output voltage/current. In addition, we integrated two PGCTs in series configuration to investigate the performance of output voltage. As shown in Additional file 1: Figure S2, the output voltage was nearly double based on the basic principle of superposition which also meant that the output voltage could be enhanced by integrating different PGCTs in serial connection modes.

**Fig. 3:**
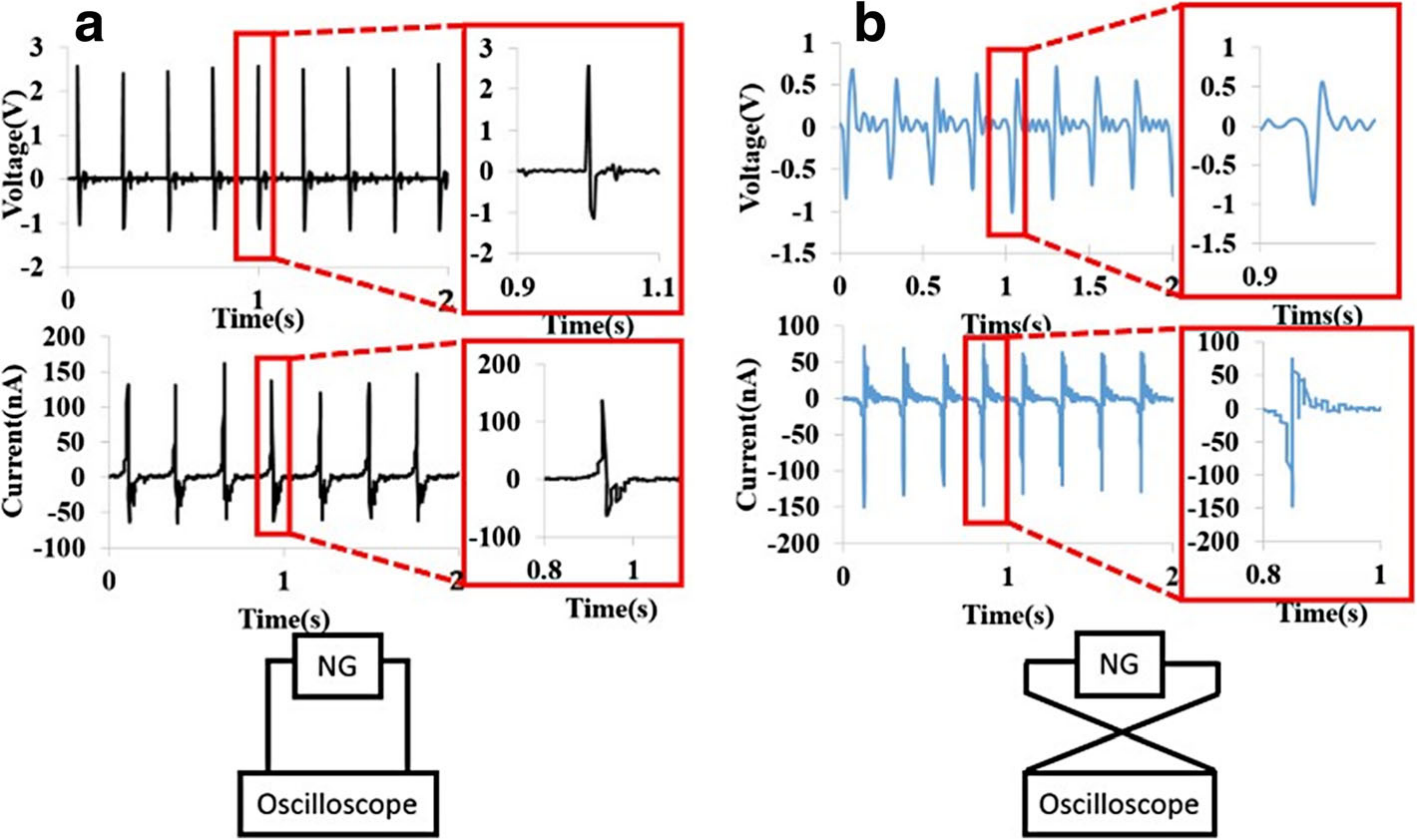
Validated polarity via forward and reverse connections measurements. The shape of output signal changed as switching the measurement polarity. **a** The peak voltage and currents generated by the PGCT of about 2.5 V and current of about 150 nA were obtained in the forward connection. **b** The output voltages and currents generated by the PGCT of about ~1 V and about ~150 nA in the reverse connection

It is crucial for the piezoelectric generator to validate polarity via a polarity test. To confirm the measured results were generated from the true piezoelectric responses instead of background or triboelectric signals. The common method to validate polarity was applied forward and reverse connections measurements. Based on the experiment, if we changed the contacts of the polarity, the shape of the response signals should be reversed immediately. While the shape of the response signals remains the same under forward and reverse connections measurements, the signal is definitely obtained from the noise or other forms instead of piezoelectric signal. The forward connection in the voltage and current measurements are depicted in Fig. 3a. The peak voltage and current in the forward connection were about 2.5 V and 150 nA, respectively, which were generated via a cantilever flapping at constant frequency (~4.5 Hz). In contrast to the forward connection, in Fig. 3b, the peak voltage and current in the reverse connection are about ~1 V and ~150 nA, respectively, which are generated via a cantilever flapping at constant frequency (~4.5 Hz) too. The enlarged insets clearly show that the shape of the response signals is reversed in the reverse connection.

Hence, the measurement result of polarity check was successfully carried out to confirm the true piezoelectric signals which were generated from the PGCT. To further validate if the PGCT does have the piezoelectric property, we collected the spectroscopic evidence of X-ray diffraction (XRD) and Fourier transform infrared spectroscopy (FTIR) in Additional file 1: Figure S3–S4, respectively. The peaks of β-phase which is majorly responsible for piezoelectricity were dominated in the XRD and FTIR results of NFES PVDF fibers [36].

**Fig. 4:**
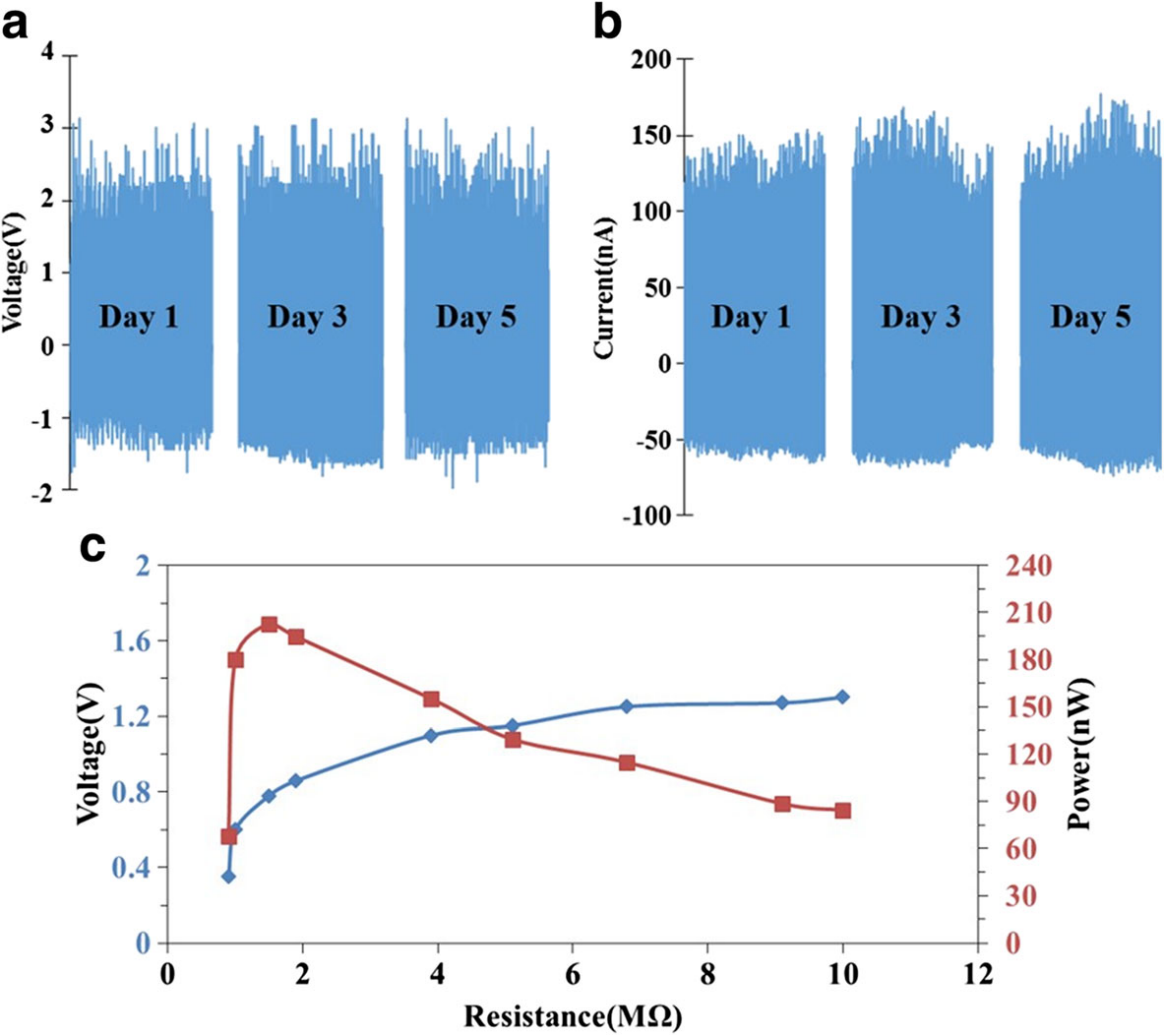
Stability tested for five consecutive days. The output (**a**) voltages and (**b**) currents of the PGCT operating at 5Hz for 10 min per day. **c** The impedance matching test of the output voltage and output power on external load resistances for the PGCT

In consideration of the practical applications of our developed devices, we further investigated the stability and robustness. In Fig. 4a, b, the PGCT was tested for five consecutive days to demonstrate the stability of output voltage and current (we collected data at day 1, 3, and 5 as representative.) at constant frequency of 5 Hz for 10 min per day. The results showed only a negligibly small variation of output performance between each day under the continuous cycles of stretching and releasing process. The highly stable power generation indicated the great stability and robust life time of the PGCT has. Correspondingly, the impedance matching test of the output voltage and output power on external load resistances for the PGCT was conducted to characterize the maximum efficiency as an energy harvester. Figure 4c exhibits the experimentally measured output voltage and power against the external load resistance. The experiment result indicated the output voltage keeps arising as the load resistance increases before the corresponding power output reaches the optimized output power of 200 nW at matched resistance of 2 MΩ. This result coincides well with previously published PVDF based harvesters with the matching resistance were in the same order of the magnitude, MΩ [21, 25].

## Results and Discussion

**Fig. 5:**
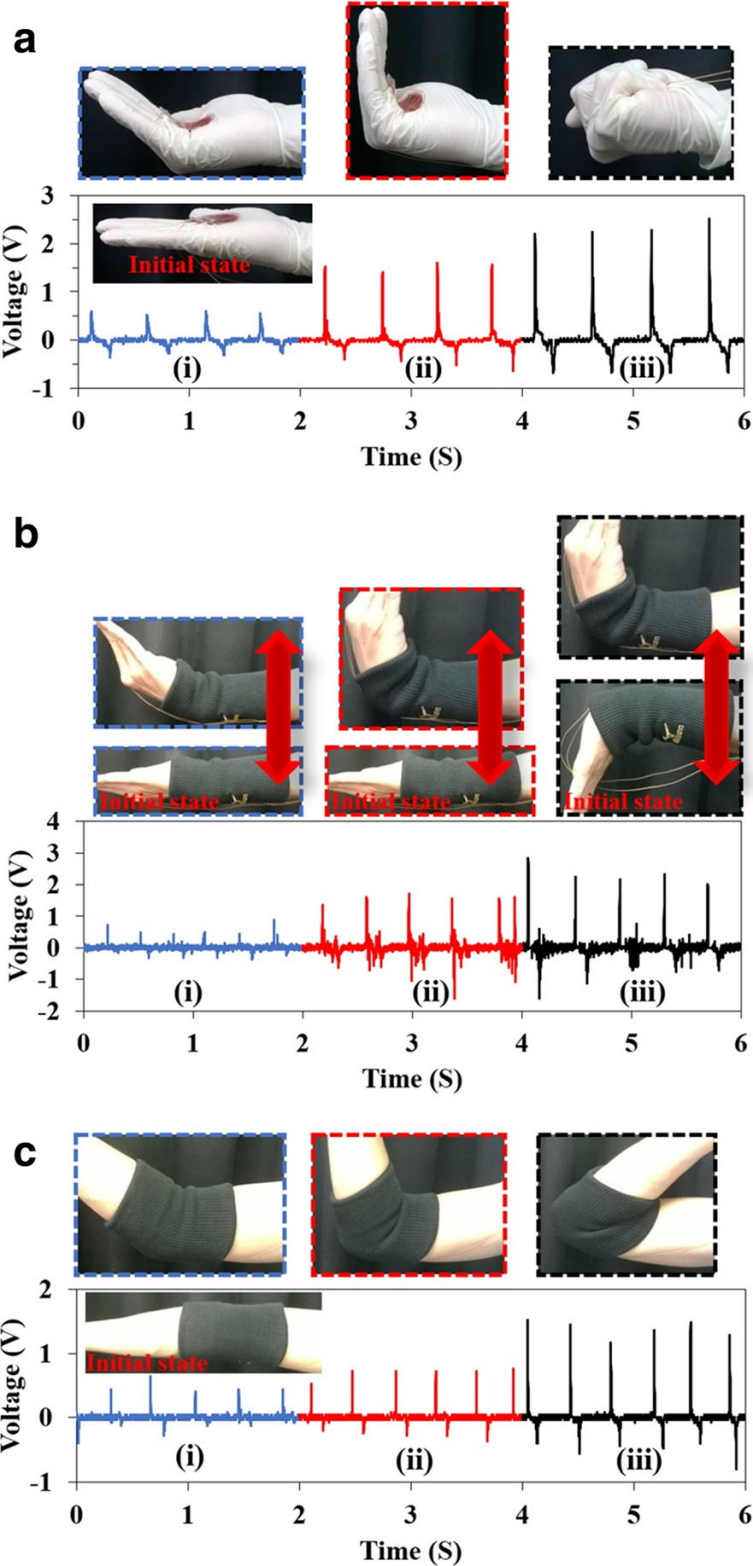
Investigated the performances of the highly flexible PGCT when acted as an active sensor under various body movements. **a** Placed the PGCT on the palm and measured the output voltage at different holding angle (*i*) 45° (*ii*) 90° (*iii*) 180° (fisted) compared to the initial state. **b** Integrated the PGCT with a wrist brace to measure the output voltage at different wrist bending angle (*i*) 45° (*ii*) 90° (*iii*) 180° as compared to the initial state. **c** Integrated the PGCT with an elbow brace to measure the output voltage at different elbow bending angle (*i*) 45° (*ii*) 90° (*iii*) 135° as compared to the initial state

The concentric circle fiber-based generator which demonstrated the good sensitivity and high conformability as a prototype of active human motion sensor is shown in Fig. 5. This super-flexible device was attached on the latex glove and operated at different holding angle as shown in Fig. 5a. The output voltages are about 0.6/1.5/2.2 V at palm holding angle (i) 45°/ (ii) 90°/ (iii) 180° (fisted) compared to the initial state, respectively. Besides, we further investigated the potential of PGCT to detect and distinguish the specific wrist/elbow movement. In Fig. 5b, the PGCT was integrated with a wrist brace as an active joint sensor to measure the output performance at different wrist bending angle. The output voltages are about 0.4/1.6/2.1 V at wrist bending angle (i) 45°/ (ii) 90°/ (iii) 180° compared to the initial state respectively. Similarly, we integrated the PGCT with an elbow brace to measure the output performance at different elbow bending angle. The output voltages are about 0.5/1/1.7 V at wrist bending angle (i) 45°/ (ii) 90°/ (iii) 135° compared to the initial state, respectively. The results demonstrate that the obtained signals are discernible between different bent angles which means that we can easily infer and identify the behavior of human joint motion from analyzing the characteristic output signals. However, the conventional cyber garment and sensor both need external power supply, combine this developed function with the naturally self-powered ability of PGCT that could be promising to acquire an active rehabilitation sensor or cyber garment without any waste of commercial battery.

## Conclusions

In summary, the purpose of this paper is to demonstrate the highly ordered and controllable concentric circle configuration of PVDF piezoelectric fibers which have the ability to harvest the mechanical energy in any deformation direction. The utilization of NFES direct-write process is a promising method to obtain massively deposited, in situ polarized piezoelectric fibers into various patterning arrays without further treatments. The massive arc piezoelectric fibers were successfully fabricated into a concentric circle configuration and show a great potential to efficiently convert mechanical energy, irrespective of the applied deformation direction. The major contribution is to resolve the inability of parallel aligned PVDF fibers to harvest energy only in parallel direction of deformation. In addition, the validated experiment showed the stable output voltage/current under different testing direction and the magnitude of output is comparable to the counterpart of parallel aligned PVDF fibers. The fully packaged device is able to produce a peak voltage of ~2.5 V and current of ~150 nA, even underwent a reliable stability test for five consecutive days. Finally, these collective consequences demonstrated that our flexible piezoelectric NMFs can be cost-effectively fabricated and easily integrated into wearable electronics such as smart cyber skin/garment, human actions monitor, joint rehabilitation evaluation, etc. We believe our innovative configuration would be beneficial to the future study of flexible and wearable electronics.

## Additional files

Additional file 1: Figure S1. Schematic of the experiment layout. The demonstrated GPFG was fixed at one end. The output voltage and current were generated via a rotating rod which driven by a commercial DC motor (RS-545SH). The induced strain can be altered by adjusting the contact position, and the actuating frequency can be easily tuned by the DC motor speed. **Figure S2.** Two PGCTs were superimposed to enhance the output voltages which. PGCT #A and PGCT #B subject to continuous stretch and release. Constructively, output voltages were basically added when two PGCT are in serial connection. All measurement data are performed when the two PGCTs operated in the same strain, strain rate, and frequency. **Figure S3.** XRD patterns of original PVDF powder (blue line), NFES PVDF fiber (red line) and conventional electrospinning PVDF thin film (green line). **Figure S4.** FTIR spectra of the PVDF powder and electrospinning PVDF fibers. The polymer solution 16 wt% PVDF, solvent (DMF:acetone with 1:1 weight ratio), 4 wt% fluorosufactant (Capstone® FS-66) was used for the electrospinning experiment. (PDF 357 kb)

## Abbreviations

direct-write NFES: Direct-write near-field electrospinning
direct-write PVDF: Direct-write Polyvinylidene fluoride
FTIR: Fourier transform infrared spectroscopy
NG: Nanogenerator
NMF: Nano/micro fibers
NWs: Nanowires
PDMS: Polydimethylsiloxane
PGCT: Piezoelectric generator with concentrically circular topography
PVC: Polyvinylchloride
PZT: Lead zirconate titanate
SEM: Scanning electron microscopy
XRD: X-ray diffraction
ZnO: Zinc oxide
