# Supplementary material for: Self-Powered Active Sensor with Concentric Topography of Piezoelectric Fibers
Source: Nanoscale Res Lett. 2017 Jan 17;12:44. doi: 10.1186/s11671-016-1786-x (PMC5241262; doi:10.1186/s11671-016-1786-x)
Supplement: Additional file 1: Figure S1. — Schematic of the experiment layout. The demonstrated GPFG was fixed at one end. The output voltage and current were generated via a rotating rod which driven by a commercial DC motor (RS-545SH). The induced strain can be altered by adjusting the contact position, and the actuating frequency can be easily tuned by the DC motor speed. Figure S2. Two PGCTs were superimposed to enhance the output voltages which. PGCT #A and PGCT #B subject to continuous stretch and release. Constructively, output voltages were basically added when two PGCT are in serial connection. All measurement data are performed when the two PGCTs operated in the same strain, strain rate, and frequency. Figure S3. XRD patterns of original PVDF powder (blue line), NFES PVDF fiber (red line) and conventional electrospinning PVDF thin film (green line). Figure S4. FTIR spectra of the PVDF powder and electrospinning PVDF fibers. The polymer solution 16 wt% PVDF, solvent (DMF:acetone with 1:1 weight ratio), 4 wt% fluorosufactant (Capstone® FS-66) was used for the electrospinning experiment. (PDF 357 kb) [file 11671_2016_1786_MOESM1_ESM.pdf]

Supplementary information

Self-powered active sensor with concentric topography of piezoelectric fibers.

Yiin Kuen Fuh[1,2], Zih Ming Huang[1], Bo Sheng Wang[1], Shan Chien Li[1]

[1]Department of Mechanical Engineering, National Central University, Taoyuan City, Taiwan.

[2]Institute of Materials Science and Engineering, National Central University, Taoyuan City, Taiwan.

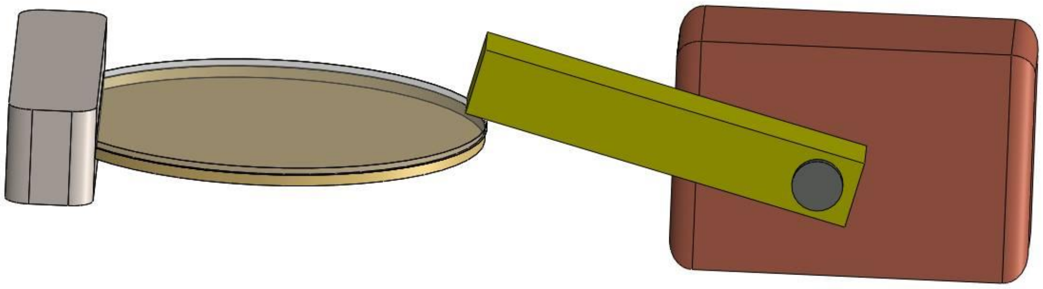

Figure S1. Schematic of the experiment layout. The demonstrated GPFG was fixed at one end. The output voltage and current were generated via a rotating rod which driven by a commercial DC motor (RS-545SH). The induced strain can be altered by adjusting the contact position, and the actuating frequency can be easily tuned by the DC motor speed.

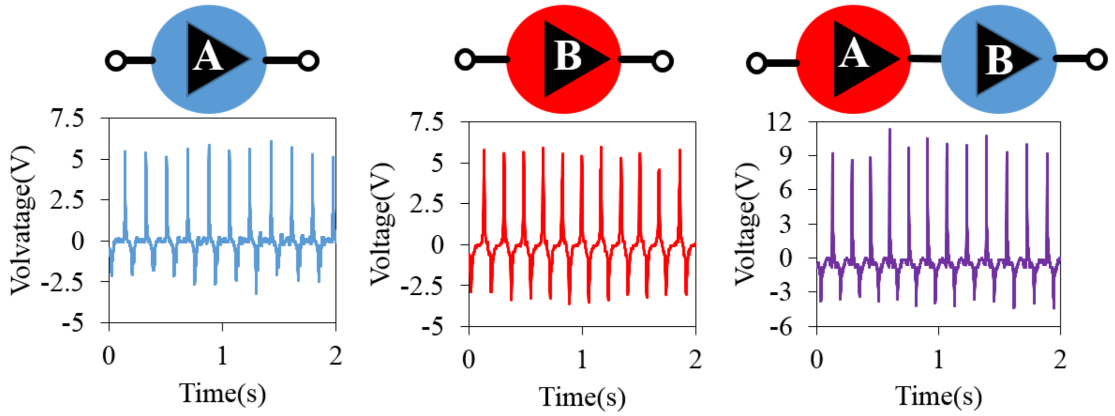

Figure S2. Two PGCTs were superimposed to enhance the output voltages which. PGCT #A and PGCT #B subject to continuous stretch and release. Constructively, output voltages were basically added when two PGCTs are in serial connection. All measurement data are performed when the two PGCTs operated in the same strain, strain rate, and frequency.

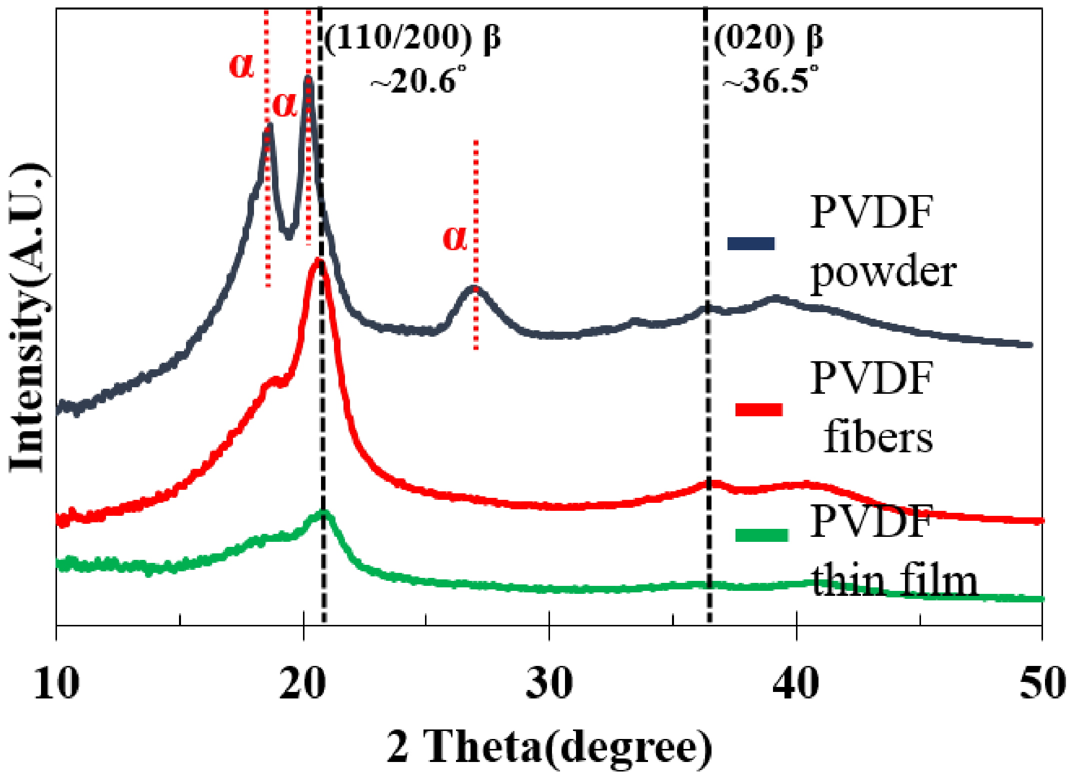

Figure S3. XRD patterns of original PVDF powder (blue line), NFES PVDF fiber (red line) and conventional electrospinning PVDF thin film (green line). XRD measurement results show two obvious diffraction peaks at $2\theta = 18.7°$, $20.2°$ and a weaker peak at $2\theta = 26.9°$ corresponding to 020, 110 and 021 reflections of the α-phase (monoclinic) crystal, respectively, are clearly observed in the PVDF powder (blue line). The NFES PVDF fiber (red line) and conventional electrospinning PVDF thin film (green line) are both observed a very strong diffraction peak at $2\theta = 20.6°$ and a weak peak at $2\theta = 36.5°$ corresponding to 110/200 and 020 reflections of the β-phase (orthorhombic) crystal, respectively. The above measurements are in good agreement with previous study on NFES electrospun nanofibers. [37, 38]

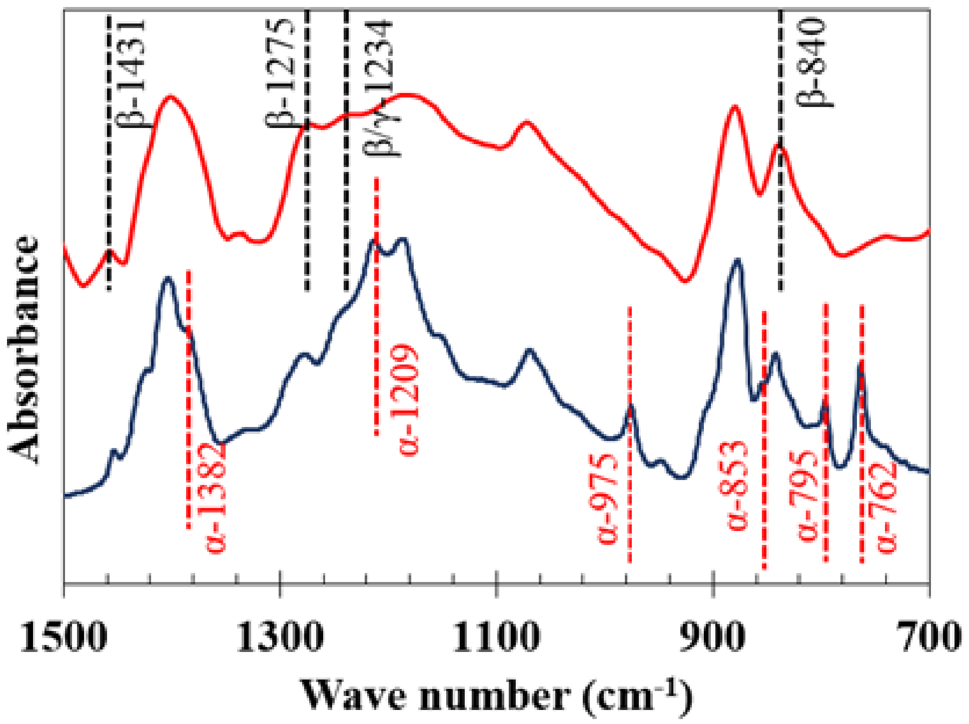

Figure S4. FTIR spectra of the PVDF powder and electrospinning PVDF fibers. The polymer solution 16 wt% PVDF, solvent (DMF:acetone with 1:1 weight ratio), 4 wt% fluorosufactant (Capstone® FS-66) was used for the electrospinning experiment. The characteristic absorption bands of the β-phase at 840, 1275, and 1431 cm-1 were observed in PVDF fiber (red line). Moreover, the characteristic absorption bands of the non-polar α-phase were observed in PVDF powder (blue line) at 762, 795, 853, 975, 1209 and 1382 cm-1.
